# The Assistive Device Situation for ALS Patients in Norway

**DOI:** 10.1155/2021/5563343

**Published:** 2021-08-18

**Authors:** Jenny Pernilla Rolland, Mari-Anne Myrberget, Tore Wergeland Meisingset

**Affiliations:** ^1^Faculty of Medicine and Health Sciences, Norwegian University of Science and Technology, 7491 Trondheim, Norway; ^2^Department of Clinical Services, St. Olav's University Hospital, 7006 Trondheim, Norway; ^3^Institute of Neuromedicine and Movement Science, Norwegian University of Science and Technology, 7491 Trondheim, Norway; ^4^Department of Neurology and Neurophysiology, St. Olav's University Hospital, 7006 Trondheim, Norway

## Abstract

**Aims:**

There are limited analytical descriptions of the assistive device situation in Norway for patients with ALS and other motor neuron diseases. This study is aimed at investigating how patients, caregivers, and healthcare professionals (occupational therapists and physiotherapists) experience the assistive device situation.

**Methods:**

Twenty-four interviews were conducted with patients with motor neuron disease, caregivers, and healthcare professionals involved in procurement and adaptation of assistive devices. Systematic text condensation was used to analyse the interviews.

**Results:**

The majority of patients and caregivers had positive experiences of follow-up by the specialist healthcare service. Several found follow-up by the primary health service to be deficient owing to inadequate expertise, continuity, and resources. Healthcare professionals reported having a proactive approach to identifying needs for assistive devices, but for various reasons, application processes were often delayed. Several patients indicated a reluctance to use assistive devices and were ambivalent regarding proactivity. The availability of assistive devices for some functional impairments was described as inadequate. Some patients felt there was too little focus on sexuality in the follow-up. The respondents had a number of suggestions for improving the assistive device situation.

**Conclusions:**

Multidisciplinary ALS teams are found to ensure follow-up expertise and continuity. Healthcare professionals wish to take a proactive approach to assistive devices, but a number of bureaucratic obstacles occur. The study findings are preliminary and should be validated through a prospective national quality registry for motor neuron diseases.

## 1. Introduction

“Motor neuron diseases” is an umbrella term for neurodegenerative disorders in which the motor division of the nervous system is selectively affected. Amyotrophic lateral sclerosis (ALS) is the most common motor neuron disease. It causes progressive paralysis of the striated musculature, but there is great heterogeneity in presentation and disease course [1]. Assistive devices and home modifications are some of the most important interventions in ALS care [2–4]. Assistive devices include medical devices designed to compensate for loss of function and improve activities of daily living. Occupational therapists play a key part in the procurement and customizing processes of assistive technology devices for patients with functional impairments and a need for facilitation. Physiotherapists also take part. Individual adaptation is necessary, creating a need for a wide range of aids [4, 5]. Clinical experience indicates that there is less satisfactory provision for some functional impairments than for others.

Follow-up of patients and caregivers by organized, multidisciplinary ALS teams has proved to enhance the quality of life and increase the survival of patients [6–8]. The goal of proactive adaptation of assistive devices is to be one step ahead of the need for aids by planning accommodations for functional limitations before they become pronounced. Proactivity is stressed as an aim in the follow-up of ALS patients [3], but how this is put into practice is not described.

Little has been written in the Norwegian or international literature about the need for and provision of assistive devices. Clinical evidence indicates that personalising assistive devices is work-intensive and complex and has varying degrees of success. This study is aimed at surveying the current assistive device situation as experienced by patients and caregivers, as well as by healthcare professionals with a particular responsibility related to adaptation of assistive devices.

## 2. Material and Method

Semistructured interviews were conducted with patients with motor neuron disease, close caregivers, and professionals in the healthcare service. All participants were followed up by ALS teams at one of four recruitment hospitals: St. Olav's University Hospital, the University Hospital of North Norway, Østfold Hospital Kalnes, and Drammen Hospital. The inclusion criterion for patients was a diagnosis of ALS or other variants of motor neuron disease. Exclusion criteria were late or terminal phase of the disease and a prior diagnosis of cognitive impairment. These limitations were set to exclude patients with a very short life expectancy and/or a physical inability to take part in interviews of this extent due to late disease stage. Patients on invasive ventilatory support were not included. Presumed eligible patients were informed of their option to take part by a neurologist or other healthcare professional in their ALS team. Final assessment of eligibility was done prior to written consent by study personal, and in case of uncertainty, ruling on eligibility was to be done by the project manager (TWM). Caregivers were recruited with the consent of the patient. If patients had difficulty communicating, their caregiver could assist during the interview. In two cases, interviews were conducted only with the patients' caregivers, as the patients themselves did not wish to be interviewed. Healthcare professionals with responsibility regarding assistive device procurement were recruited through the ALS teams at follow-up hospitals. Six were occupational therapists, and one was a physiotherapist. Two of the seven were employed in the specialist healthcare service and the others in the primary health service.

In the period August–September 2019, 24 participants were recruited for interviews. Nine were patients with motor neuron disease, eight were caregivers, and seven were healthcare professionals. No referred patients were excluded in the process. There were 14 participants from the Central Norway Regional Health Authority, six from the South-Eastern Norway Regional Health Authority, and four from the Northern Norway Regional Health Authority. The average age of the patients was 61.6 years (range 45–74), the average duration of disease was 7.0 years (0.83–21), and gender distribution was even. The median distance to follow-up hospital was 9.5 kilometres (3–160). The Revised ALS Functional Rating Scale (ALSFRS-R) was used as a measure of function. The scores of eight patients were obtained, as two patients were not interviewed directly and one patient was not scored. The median ALSFRS-R was 25 (range 10–42) (Table 1).

The interview guide was developed by the study group (JPR, MAM, and TWM). It was structured similarly, but with some necessary adjustments in wording for the three target groups. The interview guide addressed issues relating to follow-up and own experience with assistive devices and any limitations on their provision. The participants were urged to reflect freely in response to the questions. With the aim of capturing free and unrestricted reflections concerning assistive devices, we intentionally provided no definitions. Thus, patients could include whatever they considered relevant, for instance medical devices such as noninvasive ventilation. Within the interview guide, the interviewer had the liberty to explore answers further. The same interview moderator (JPR, a senior medical student and not part of the team delivering services to patients) conducted all interviews. The interview guide was piloted on the first participants. The guide was revised during the process by the study group. No major changes were made during the revisions. The questions were thus asked fairly equally for all patients throughout the interview process. All interviews were conducted face-to-face in order to best capture the participants' expressions and feelings. Audio recordings were made of the interviews, which were transcribed subsequently. Systematic text condensation was used in analysing the data [9]. This is a qualitative analysis strategy consisting of four stages: (1) reading through the material to form an overall impression and picking out themes; (2) identifying units of meaning and coding of these; (3) dividing up code group contents into subgroups, condensing the content, and identifying quotes; and (4) rewriting condensates into an analytical text with generalized descriptions of the assistive device situation for motor neuron diseases.

An analytical log documented the development of code groups and categories. The project has been approved by the Regional Committee for Medical and Health Research Ethics (ref. 2018/2164).

## 3. Results

### 3.1. Proactivity and Procurement Processes

Healthcare professionals had a clearly proactive attitude to identifying needs for assistive devices. This is applied generally to both the primary and the specialist healthcare services in all health regions. The reason for anticipating needs was the importance of practicing on and becoming familiar with assistive devices, particularly when procuring communication devices, and given expectations of delays in the delivery process. Patients were more ambivalent than healthcare professionals with respect to proactivity. Patients believed it was important to be one step ahead of disease progression, but did not want too much information about expected future needs. Caregivers, on the other hand, wanted information about expected progression in order to be prepared. Individual adaptation of information provided to patients and caregivers, respectively, was important.

The way the passage of time was experienced from when a need was identified until assistive devices were delivered varied widely among the interview respondents. Healthcare professionals stated that it often took a long time for assistive devices to reach patients. The time taken appeared to vary, depending on the type of assistive device that was ordered, from one municipality to the next. Practical obstacles, such as large distances to the follow-up hospital and the assistive technology centre, and patient-specific factors such as expectations regarding own disease progression appeared to add to delays in application processes, in addition to paperwork and bureaucracy. Some patients spoke of reluctance to use assistive devices because of a desire to manage on their own as long as possible. This reluctance was mentioned by patients, caregivers, and healthcare professionals alike as a factor behind delays in getting assistive devices into place. Rapid disease progression presented a particular challenge in procurement of assistive devices, in terms of both identification of needs and speed of delivery.

“The recliner chair was supposed to be specially adapted, but it doesn't work. The patient's disease has progressed so rapidly that he is unable to use the recliner chair. [...] The way it is with ALS, by the time the assistive device has arrived, the disease may have progressed and the patient's condition substantially changed.”

### 3.2. Expertise and Continuity

Several patients and caregivers experienced follow-up of the assistive devices provided in different ways and pointed out deficiencies. Some patients found that they received assistive devices they did not need. They wanted more inclusion in the procurement process to avoid this and more training in the use of the assistive devices. Their experiences of the specialist and primary healthcare services varied. Both patients and caregivers found follow-up from the specialist healthcare service at the hospital to function satisfactorily, and that patients' needs for assistive devices were well provided for. Multidisciplinarity and a high level of expertise in the ALS teams and a low threshold for contacting them were suggested as possible reasons for this. However, several thought there was room for improvement in follow-up by the primary healthcare service. Lack of expertise and experience with ALS were mentioned, along with poor capacity, limited resources, and lack of continuity because of a high turnover rate among healthcare workers and many persons to relate to in the home care service. Caregivers found it frustrating to have to teach healthcare workers how to use the assistive devices.

“Sometimes people are on holiday or sick leave. Perhaps that's the worst part, if the municipality doesn't provide a replacement. It's frustrating. The municipality should have taken steps, but the financial aspect probably comes into it.”

Caregivers thought follow-up in the primary health service functioned well if home care service employees could readily contact the specialist healthcare service for guidance and advice. Caregivers found that in cases where this cooperation functioned smoothly, it led to rapid identification of needs for assistive devices.

### 3.3. Limitations in the Supply of Assistive Devices

For several patients, the lack of assistive devices for upper extremities was the greatest restriction on daily living. Whereas there are several good assistive devices to compensate for impaired lower extremity functioning, half of the respondents commented on deficiencies in the availability of assistive devices for upper extremities. This applied to both gross and fine motor functions of the arms. Assistive devices for dressing and eating were both inadequate, meaning that patients were dependent on help from others. Eating devices were described as complicated to control and created a great deal of mess for the patient. Some managed with adaptations, while others chose rather to be fed.

“There is a lot that I would have liked to carry on doing. Especially handicrafts – I've been doing that all my life. I miss it. Everything from crocheting, knitting and painting, to scratching my nose. We use our hands for so much.”

Two patients spoke spontaneously of lack of information concerning available assistive devices for maintaining normal cohabitation and intimacy. The respondents both had slow disease progression and were younger than the study average.

### 3.4. Potential for Improvement

Patients and caregivers had a number of ideas about potential improvements in follow-up of assistive technology. They wanted a better flow of information regarding devices, specific recommendations, improved logistics with an overview of what assistive devices were available, and more trying out and testing. Patients wanted to evaluate assistive devices and for their feedback to reach the manufacturers. They also wanted to see other people's evaluations before procurements were made. The caregivers were of the view that everyone in the care service should have the same training in the use of assistive devices, to ensure competence and prevent caregivers having to provide the training.

## 4. Discussion

The purpose of this study was to survey the way the current situation with respect to assistive technology devices for motor neuron diseases in Norway is experienced by patients and their caregivers, as well as by the healthcare professionals who work most closely with adaptation of assistive devices. The experiences of patients and caregivers regarding follow-up were found to vary. The respondents had different perspectives on identifying needs for assistive devices. Both the actual availability of assistive devices and the flow of information between healthcare professionals and patients and caregivers were found to present challenges.

### 4.1. Strengths and Weaknesses

Qualitative methods are suitable for providing insight into individuals' own experiences through open-ended questions that can capture both nuances and a multiplicity of answers [10]. Because of the complexity of the issue, a qualitative method was regarded as appropriate. As limited prior data to instruct the design of this study was identified, the interview guide was intentionally designed to allow a high degree of free reflection. For instance, definitions on what constitute an assistive device were not provided. Twenty-four interviews were conducted, which is a large number for a qualitative study [10]. The large number was intended to ensure sufficient variation in the sample, both geographic and phenotypic. The interviews reflected the situation of 11 patients, of different ages and with different phenotypes, in early and established stages of the disease. We had participants from three health regions with varying distances to follow-up hospitals and assistive technology centres, different follow-up systems, and living in both urban and rural areas. As a result, the backgrounds and experience of the participants varied. Where possible, caregivers were interviewed without the patient present.

Patients and caregivers spoke consistently of good follow-up by the specialist health service, but the fact that all patients had outpatient follow-up from hospitals with established ALS teams must be borne in mind when interpreting this finding. The study was not designed to compare the specialist and primary health services. At the same time, patients in more than one health region were found to have overlapping problems with respect to follow-up by the primary health service. These relate especially to the continuity of follow-up and expertise in motor neuron disease. One relevant measure would be to increase contact points between ALS teams and the municipal health service, particularly with a view to building expertise in the primary health service and contributing to continuity in the follow-up of the individual patient.

### 4.2. Time Aspects and Proactive Approaches

The healthcare professionals in this study had a clearly proactive approach to identifying needs for assistive devices, but experienced long delivery times for several types of assistive devices, particularly where customization was involved. The result was that function loss might have progressed to the extent that by the time the assistive devices were delivered, they were no longer adequate. Studies from Germany and the Netherlands have pinpointed delays in procurements, bureaucratic obstructions, and unsuccessful deliveries as significant obstacles in the procurement of assistive devices in these countries [3, 11, 12]. Our study indicates a need to improve the efficiency of procurement processes in the Norwegian system as well. Guidelines at national level, including specific recommendations to guide healthcare professionals in selecting assistive devices, may contribute to this and help ensure that individual patients receive appropriate assistive devices. Specific recommendations will be of particular help to healthcare professionals with limited experience and expertise in ALS.

Caregivers and patients were more ambivalent regarding proactivity. Patients did not want too much information about anticipated future needs, while caregivers wished to be prepared for disease progression. This makes it particularly demanding to provide a good flow of information. A qualitative study from Oslo University Hospital showed that patients felt that the provision of information to caregivers was not good enough [13]. Our study finds that there is still a need for more individually tailored information for both patients and their caregivers.

### 4.3. Unmet Needs

The currently available supply of assistive devices provides poor compensation for several functional impairments. In our study, a desire was expressed for better assistive devices to compensate for impairments of the fine motor skills of the hands and functional assistive devices for dressing and for eating. The loss of hand and arm function is a primary cause of functional impairment in ALS. It makes activities of daily living such as getting dressed and maintaining personal hygiene difficult and can restrict the patient's independence already at an early stage of the disease course [14]. Differences in functional impairment from one person to the next mean that flexible control mechanisms are required. There is a greater need for more individual adaptation of assistive technology devices and their control mechanisms.

Sexual function is not affected directly by ALS, but impairment of motor functions may make intimacy difficult [15, 16]. Two patients spoke spontaneously of the shortage of assistive devices available for maintaining a normal sexual relationship with a partner. They knew little about what was available and would have liked this subject to have been broached by healthcare professionals. The patients in question were younger, and the duration of their disease was longer than the study average. Several earlier studies have pointed out that sexuality is followed up to only a very limited extent by healthcare professionals [15–17]. Questions about sexual needs tend not to be put to ALS patients [18]. A stronger focus on how motor neuron diseases affect cohabitation and intimacy appears important for ensuring that the healthcare service meets patients' needs in this domain.

## 5. Conclusions

ALS patients, their caregivers, and healthcare professionals experience the current assistive device situation in a variety of ways, depending on the type of functional impairment, the progression rate of the disease, and the geographical location of residence. Some experience complicated procurement processes and bureaucratic delays, .(...)practical obstacles, and delays due to patient-specific factors. Healthcare professionals want proactive adaptation of assistive devices, whereas patients may be ambivalent at times. Procurement processes are reported inefficient, and national guidelines with recommendations concerning assistive devices are suggested. The focus should be on the potential and resources of the individual, with the aim of achieving independence, active participation, and the highest possible functioning. The results in this study should be considered as preliminary and needs validation for instance through a prospective, national quality registry for motor neuron diseases.

## Figures and Tables

**Table 1:** Patient characteristics.

Patient characteristics	Total (*N* = 11^a^)
Average age, years (range)	61.6 (45–74)
Average duration of disease, years (range)	7.0 (0.83–21)
Sex, *n* (%)	
Male	6 (54.5)
Female	5 (45.5)
Median ALSFRS-R^b^ (range)	25 (10–42)

^a^Nine patients were interviewed directly, while two patients were described by their caregivers in the absence of a patient interview. ^b^Function score for eight patients. %: percent; *N*: number; ALSFRS-R: Amyotrophic Lateral Sclerosis Functional Rating Scale-Revised.

## Data Availability

Interview transcripts will not be able to be published due to the possibility of identifiability. Access to raw data is restricted due to ethical concerns regarding third-party rights and privacy. All other information data is embedded within the manuscript. Raw data can be made available (...) can be made available to the editor for confirmation purposes.
