# Erratum to “The Assistive Device Situation for ALS Patients in Norway”

**DOI:** 10.1155/2022/9807421

**Published:** 2022-10-25

**Authors:** Jenny Pernilla Rolland, Mari-Anne Myrberget, Tore Wergeland Meisingset

**Affiliations:** ^1^Faculty of Medicine and Health Sciences, Norwegian University of Science and Technology, 7491 Trondheim, Norway; ^2^Department of Clinical Services, St. Olav's University Hospital, 7006 Trondheim, Norway; ^3^Institute of Neuromedicine and Movement Science, Norwegian University of Science and Technology, 7491 Trondheim, Norway; ^4^Department of Neurology and Neurophysiology, St. Olav's University Hospital, 7006 Trondheim, Norway

In the article titled “The Assistive Device Situation for ALS Patients in Norway” [1], the authors wish to correct two typographical errors in Conclusion and Data Availability that were introduced while in the production stages.

The errors are as follows:

1. In Conclusion, “Some experience complicated procurement processes and bureaucratic delays,.(…)practical obstacles, and delays due to patient-specific factors” should read as

“Some experience complicated procurement processes and bureaucratic delays, practical obstacles, and delays due to patient-specific factors”

(2) In Data Availability, “Raw data can be made available (...) can be made available to the editor for confirmation purposes” should read as

“Raw data can be made available to the editor for confirmation purposes”
